# Diet in Disease

**Published:** 1893-07-01

**Authors:** Ernest Hart

**Affiliations:** Bachelier-és-Sciences-es-Lettres (réstreint), formerly Student of the Faculty of Medicine of Paris, and of the London School of Medicine for Women


### DIET IN DISEASE.

By MRS. ERNEST HART, Bachelier-ès-Sciences-es-Lettres (rêstreint), formerly Student of the Faculty of Medicine of Paris, and of the London School of Medicine for Women.

### XXV.—ON THINNING AND FATTENING.

THE accumulation of fat in the body is a very general condition after the age of forty. Those who have read the previous paper will not be surprised to learn that this accumulation of fat is due to the intake of food being larger than is necessary for the requirements of the body. "Why, I am a very small eater, and yet I grow fat," is the indignant exclamation; for there is nothing people resent so much as being told that they eat too much. The inexorable fact, however, remains that if you grow fat, you either take too much food in bulk, or too much food of a certain kind, that is of a fattening kind. This excess may be every day exceedingly small, and yet in the course of a year, or a series of years, it results in transforming a once graceful figure into an unwieldy shape, and an active energetic person into one to whom movement and exercise are repugnant. Dr. Burney Yeo has shown in a very practical way how a large accumulation of fat may take place in a few years from a very small cause. He instances the case of a person who daily takes in excess of his wants half an ounce (about two lumps) of sugar. This sugar not being refined and burnt off is converted into fat, and stored up in his body as such. This daily consumption of sugar, and this daily storage of fat, will result in one year in increasing his weight eleven pounds, and in five years in raising it no less than four stones. From this example it is seen how in middle age, when metabolism is slow, a small excess of fattening foods will lead to obesity by imperceptible degrees. They rarely have the same effect in youth when the body is able to consume and get rid of much larger quantities of food. There are, it is true, certain cases of obesity which are pathological instead of physiological; these are generally associated with anæmia, fatty degeneration of the heart, leading to imperfect oxidation in the tissues, or with hysteria. Such cases are not within the range of this paper, and should be treated by the physician as symptoms of some grave disease.

**Treatment.**—In the treatment of ordinary obesity there are certain definite principles to be followed:—

1. To oblige the body to feed for a while on itself, and to consume its own fat;
2. To prevent the re-accumulation of fat.

Both these conditions can be accomplished by diet.

Before commencing the dietetic treatment of obesity the patient should undergo a careful physical examination by a physician to ascertain if the organs, particularly the heart and kidneys, are sound. Some of the systems in vogue for reducing fat tax these organs severely, and if there are any signs of incompetency or disease of the heart or kidneys, the system adopted should be modified accordingly. It will be remembered, that when explaining the action of hydrocarbons and carbo-hydrates in the body, it was stated that carbo-hydrates — *i.e.*, starchy and sugar foods — are the substances out of which adipose tissue is manufactured; but that, as in the case of fattening pigs, the production of fat in the body is much more rapid if fatty foods are taken in combination with starchy foods. Thus it was shown that pigs fatten much more rapidly on meal and greasy pig-wash than on meal alone. From these facts we infer that the ordinary mixed diet of adults, consisting of meat, fat, and farinaceous foods, is, after the period of youth and activity is passed, liable to cause an excessive deposit of fat. The indication is, therefore, to cut off the farinaceous foods, and, according to some authorities, to cut off the fatty foods as well. It is not, however, sufficient to reduce the consumption of fatty and farinaceous foods; the intake of food, and more especially of fluid food, must be reduced generally. It is useless for the fat person to say, "I will not diet myself, I will take more exercise." If he increases exercise he will probably increase appetite, and consequently the intake of food. He must increase exercise and decrease the intake of food, being willing to suffer even for a time the pangs of hunger till the body learns to feed upon itself, and habit has modified appetite.

There are three typical and well-known methods of treating obesity. One has a wide popularity, and is known by the name of its inventor, Banting. This system consists in increasing the amount of albuminous or meat foods, and of greatly decreasing both the fat and starchy foods. The second is that advocated by Ebstein, the German physician, in which the albuminous foods are greatly diminished, and the starchy foods still more so, but the amount of fat taken is normal. The third is that practised by Oertel, in which the albuminous foods are increased, and the fats and carbo-hydrates reduced to about a fourth of the normal. All the methods agree in one particular, that is, in reducing the total bulk of the food taken. Thus, while the normal amount of food consumed by a healthy man will amount in grammes to 618, Banting would reduce this to 260, Ebstein to 235, and Oertel to 280. The following table (taken from Dr. Burney Yeo's book) clearly states the kind and quantities of food allowed:—

| | Albuminates. | F ts. | Carbo-hycrates. |
|---|---|---|---|
| Normal average of grammes | 130 ... | 84 ... | 404 |
| Banting ... ... ... | 170 ... | 10 ... | 80 |
| Ebstein ... ... ... | 100 ... | 85 ... | 50 |
| Oertel ... ... ... ... | 155 179 ... | 25·40 ... | 70·10 |

The Banting *régime* was as follows:—

BREAKFAST at 9 a.m. to consist of 5 to 6 oz. of animal food —meat (except pork and veal) or boiled fish; a little biscuit, or 1 oz. of dry toast—6 or 7 oz. of solids in all. A large cup of tea or coffee (without milk or sugar)—9 oz. of liquids.

DINNER at 2 p.m.—Fish or meat (avoiding salmon, eels, herrings, pork, and veal), 5 to 6 oz., or any kind of poultry or game. Any vegetables, except potato, parsnips, beetroot, turnips, or carrot. Dry toast 1 oz. Cooked fruit, unsweetened. Good claret, sherry, or Madeira, 10 oz. Total of solids 10 to 12 oz.

TEA, 6 p.m.—Cooked fruit, 2 to 3 oz., a rusk or two; 2 to 4 oz. of solids; 9 oz. of tea without sugar or milk.

SUPPER, 9 p.m.—Meat or fish, as at dinner, 3 to 4 oz. Claret or sherry and water, 7 oz.

On this diet Mr. Banting reduced himself in one year from 14st. 6lb. to 11st. 2lb. There is, it will be noted, the greatest possible limitation of carbohydrates and fats, they being reduced from the normal of about 500 to less than 100. The large amount of meat taken in the Banting *régime* is to some people extremely distasteful, and in the cases where the kidneys are not healthy, or where there is a tendency to rheumatism, this severe dietary may be actually injurious.

The Ebstein *régime* is based on the theory that it is the starchy and saccharine foods, not the fats, which form fat, and that it is not necessary to reduce the latter. In fact, Ebstein contends that the ingestion of fat is useful in curing obesity, if combined with a greatly reduced food supply in albuminates and carbohydrates, as fat abates appetite and diminishes thirst. About half the usual amount of meat is allowed. As seen from the following dietary the Ebstein diet is very meagre, although it does contain butter and fat.

Breakfast (6 a.m. in summer, 7.30 in winter).—White bread, well toasted (rather less than 2 oz.) and well covered with butter. Tea, without milk or sugar, 8 or 9 oz.

Dinner, 2 p.m.—Soup made with beef marrow. Fat meat with fat gravy, 4 to 5 oz. A moderate quantity of one of the vegetables allowed, namely, asparagus, spinach, cabbage, peas, and beans. Two or three glasses of light white wine. After this meal a large cup of tea, without milk or sugar.

Supper, 7.30 p.m.—An egg, a little roast meat with fat, about an ounce of bread well covered with butter, a large cup of tea, without milk or sugar.

In the Oertel system the maintenance of the general health is carefully considered while the fat of the body is being reduced. Steady walking exercise to strengthen the muscles of the heart is insisted upon, and walking slowly uphill and going upstairs are especially advocated. The quantity of fluid drunk is diminished, while perspiration is promoted by baths; the normal condition of the blood maintained, and wasting of the muscles prevented by an albuminous diet. The diet may be as follows:—

Morning.—One cup of coffee or tea, with a little milk, altogether about 6 oz. Bread about 3 oz.

Noon.—3 to 4 oz. of soup; 7 to 8 oz. of roast or boiled beef, veal, game, or not too fat poultry, salad or a light vegetable, a little fish (cooked without fat) if desired, 1 oz. of bread or farinaceous pudding (never more than 3 oz.), 3 to 5 oz. of fruit, fresh preferred, for dessert. It is desirable at this meal to avoid taking fluids; but in hot weather, or in the absence of fruit, 6 to 8 oz. of light wine may be taken.

Afternoon.—The same amount of coffee or tea as in the morning, with at most 6 oz. of water; 1 oz. of bread occasionally.

Evening.—One or two soft-boiled eggs, 1 oz. of bread. Salad and fruit; 6 to 8 oz. of wine, with 4 or 5 oz. of water.

It will be noticed that fluid is either forbidden at meals or taken in very small quantities. There is no doubt that taking soup and large quantities of fluid with food increases obesity.

None of the rigid dietaries given above could be followed by all persons alike; the amount of food necessary for a large active person would be excessive for one of small stature and indolent habits. Each case must be treated more or less on its merits, remembering always that there are certain broad principles to be followed. Decrease the total amount of food taken, and strictly limit the amount of drink; cut off all sugar, beer, and spirits; eat no potatoes, bread puddings or pastries; skim all milk, take no soups or fancy dishes; take a fair amount of roast or boiled meat, with fish, game, poultry, and eggs, as well as vegetables and fruit. Take steady exercise, and promote the action of the skin.

A little personal experience may be useful after so much theory, and it may be interesting to my readers who suffer from too great an abundance of fat to learn how I put theory into practice and reduced my weight 15 lbs. in three weeks. This result was obtained at Carlsbad, and the régime was as follows: Rose at six, took three tumblers of hot sprudel water, walking for about twenty minutes between each glass. Breakfast at eight, consisting of one or two small crescents of bread and a boiled egg. On alternate mornings a vapour bath with cold douche, or general massage of the body. Dinner at one o'clock, consisting of a small amount of fish and meat, or poultry, with green vegetables; no potatoes or sweets. In the afternoon a walk of from six to eight miles up the hills in a flannel dress. Supper at seven consisting of a poached egg or a small cut of cold meat. There is no doubt that I suffered from constant hunger on this limited diet, but under it my weight steadily diminished, and a feeling of lightness and well-being took the place of previous heaviness. Continuing the diet after I left Carlsbad, I lost another six pounds, and it was some years before the tendency to increase in weight showed itself again. I am quite certain that no one need fear becoming a ponderous size, a source of discomfort to themselves and of disagreeable impressions to others, if they checked the beginning of obesity by suffering the small inconvenience of submitting to a restricted diet for a time.

